# Brain Inflammation and Intracellular α-Synuclein Aggregates in Macaques after SARS-CoV-2 Infection

**DOI:** 10.3390/v14040776

**Published:** 2022-04-08

**Authors:** Ingrid H. C. H. M. Philippens, Kinga P. Böszörményi, Jacqueline A. M. Wubben, Zahra C. Fagrouch, Nikki van Driel, Amber Q. Mayenburg, Diana Lozovagia, Eva Roos, Bernadette Schurink, Marianna Bugiani, Ronald E. Bontrop, Jinte Middeldorp, Willy M. Bogers, Lioe-Fee de Geus-Oei, Jan A. M. Langermans, Ernst J. Verschoor, Marieke A. Stammes, Babs E. Verstrepen

**Affiliations:** 1 Biomedical Primate Research Centre (BPRC), 2288 GJ Rijswijk, The Netherlands; ingrid.philippens@crl.com (I.H.C.H.M.P.); boszormenyi@bprc.nl (K.P.B.); wubben@bprc.nl (J.A.M.W.); fagrouch@bprc.nl (Z.C.F.); nikki.van.driel@ddl.nl (N.v.D.); a.mayenburg@hotmail.com (A.Q.M.); ntiana_92@hotmail.com (D.L.); bontrop@bprc.nl (R.E.B.); middeldorp@bprc.nl (J.M.); bogers@bprc.nl (W.M.B.); langermans@bprc.nl (J.A.M.L.); stammes@bprc.nl (M.A.S.); b.verstrepen@erasmusmc.nl (B.E.V.); 2 Department of Pathology, Amsterdam UMC, 1081 HV Amsterdam, The Netherlands; eva.roos@amsterdamumc.nl (E.R.); b.schurink@amsterdamumc.nl (B.S.); m.bugiani@amsterdamumc.nl (M.B.); 3 Department of Biology, Theoretical Biology and Bioinformatics, Utrecht University, 3584 CS Utrecht, The Netherlands; 4 Department of Radiology, Leiden University Medical Center, 2333 ZA Leiden, The Netherlands; l.f.de_geus-oei@lumc.nl; 5 Biomedical Photonic Imaging Group, University of Twente, 7522 ND Enschede, The Netherlands; 6 Department Population Health Sciences, Faculty of Veterinary Medicine, Utrecht University, 3584 CM Utrecht, The Netherlands

**Keywords:** SARS-CoV-2, macaques, neuroinflammation, COVID-19, positron emission tomography-computed tomography, α-synuclein

## Abstract

SARS-CoV-2 causes acute respiratory disease, but many patients also experience neurological complications. Neuropathological changes with pronounced neuroinflammation have been described in individuals after lethal COVID-19, as well as in the CSF of hospitalized patients with neurological complications. To assess whether neuropathological changes can occur after a SARS-CoV-2 infection, leading to mild-to-moderate disease, we investigated the brains of four rhesus and four cynomolgus macaques after pulmonary disease and without overt clinical symptoms. Postmortem analysis demonstrated the infiltration of T-cells and activated microglia in the parenchyma of all infected animals, even in the absence of viral antigen or RNA. Moreover, intracellular α-synuclein aggregates were found in the brains of both macaque species. The heterogeneity of these manifestations in the brains indicates the virus’ neuropathological potential and should be considered a warning for long-term health risks, following SARS-CoV-2 infection.

## 1. Introduction

Severe acute respiratory syndrome coronavirus 2 (SARS-CoV-2) predominantly affects the respiratory organs, but over 30% of the hospitalized COVID-19 patients also suffer from neurological manifestations, including loss of smell or taste, delirium, diminished consciousness, epilepsy, and psychosis [1]. Furthermore, SARS-CoV-2 has been associated with cases of meningitis, encephalitis, and Guillain-Barré syndrome [2]. In addition, several case studies reported patients with COVID-19 who developed clinical parkinsonism within 2–5 weeks after SARS-CoV-2 infection [3,4,5,6].

Postmortem analysis of brain tissue from patients who died from severe COVID-19 demonstrates neuropathological changes, such as increased glial activation and the infiltration of T-cells [7,8]. Hence, there is growing concern about the long-term consequences for people who experienced only a mild, or even an asymptomatic, infection. At this point, however, it is poorly understood whether neuropathological changes are also present after a mild-to-moderate disease. To address this issue, we studied the brains of SARS-CoV-2-infected macaques.

Different animal models have been applied to investigate the neuroinvasive properties of SARS-CoV-2, as well as the virus’s potential to cause neuropathology [9,10]. SARS-CoV-2 infection-induced meningoencephalitis in human ACE2-transgenic mice within 2 days post-infection (dpi) and viral antigens were detected in various brain areas [11]. Studies using K18-hACE mice and hamsters also evidenced SARS-CoV-2 in the brain and confirmed the inflammatory processes that led to neuronal damage [12,13]; neuropathology was also seen in deer mice [14]. Non-human primates (NHPs), in particular macaques, are highly relevant models for COVID-19 research [15], but NHP studies describing SARS-CoV-2 neuroinvasion and neuropathology are limited. Choudhary et al. [16] describe viral encephalitis in a macaque euthanized at 7/8 dpi, but no viral RNA was detected. Intranasal inoculation of rhesus macaques led to neuroinflammation in the CNS and viral RNA detection in the nasal mucosa and CNS [17]. However, these animal studies have focused on the first two weeks of SARS-CoV-2 infection, which corresponds to the acute, viremic phase of infection in humans, in which various neurological symptoms, such as loss of smell and taste, appear. Yet, in many patients, symptoms persist or even worsen after the viremic phase, eventually leading to post-acute consequences of SARS-CoV-2 or long-term COVID. We focused our research on the post-acute phase of the SARS-CoV-2 infection in the macaque model, two to six weeks after experimental infection.

Four male rhesus and four male cynomolgus macaques were exposed to SARS-CoV-2 and developed, after infection, mild-to-moderate pulmonary disease symptoms [18]. In this manuscript, we report on the brains of the previous study subjects, which were systematically dissected and monitored for the presence of the virus, evidence of neuroinflammatory processes, and accumulation of the proteins related to neurological disorders [19].

## 2. Materials and Methods

### 2.1. Animals and SARS-CoV-2 Infection

The animals and infection study were previously described in detail [18]. Relevant parameters are summarized in Table 1. In brief, four male rhesus macaques (*Macaca mulatta*) and four male cynomolgus macaques (*Macaca fascicularis*) received 10^5^ TCID_50_ of SARS-CoV-2 (strain BetaCoV/BavPat1/2020) via the intratracheal and intranasal route. Viral shedding was observed in all animals, and all animals seroconverted for SARS-CoV-2-specific IgG within 17 days pi. Computed tomography (CT) revealed mild-to-moderate pulmonary disease. None of the animals showed overt COVID-19 clinical signs, other than occasional sneezing. The research protocol was approved by the appropriate national authorities (CCD, Central Committee for Animal Experiments; license number AVD5020020209404) and institutional Animal Welfare Body (AWB) (in Dutch: Instantie voor Dierenwelzijn, IvD). To assess the potential neurological impact of the virus, the brains were collected five to six weeks after SARS-CoV-2 infection. For comparison of the histological findings, we evaluated brains from age-matched uninfected macaques from the BPRC biobank. The details of the animals used as a control for histology, and the controls for PET-CT are summarized in Supplementary Table S1.

### 2.2. Positron Emission Tomography-Computed Tomography of the Brain

Positron emission tomography (PET)-CT data were acquired using a multiscan large field-of-view extreme resolution research imager (LFER) 150 PET-CT (Mediso Medical Imaging Systems Ltd., Budapest, Hungary), as described before [20]. In brief, animals were fasted overnight to decrease glucose levels below 10 mmoL/L. Sedation was induced IM with ketamine (10 mg/kg) (ketamine hydrochloride; Alfasan Nederland BV, Woerden, The Netherlands), combined with medetomidine hydrochloride (0.05 mg/kg) (Sedastart; AST Farma B.V., Oudewater, The Netherlands), and maintained with isoflurane. Thirty minutes after the IV injection of approximately 100 MBq of ^18^F-FDG (GE Healthcare, Leiderdorp, The Netherlands), a single field-of-view PET of the head is acquired for 15 min. Data was analyzed in VivoQuant 4.5 (Invicro, Boston, MA, USA). Based on repeatability and parameters for the correct interpretation of the results, a standardized uptake value (SUV) ratio between the pituitary gland and brain was used for robustness. The ratios were calculated for both the average SUV (SUV_mean_) in a region of interest and maximum average SUV within a 1-mm^3^ spherical volume SUV (SUV_peak_). Threshold levels were defined as SUV_mean_ ratio ≥ 1.5 and SUVpeak ratio ≥ 1.0.

### 2.3. Brain Tissue Sampling

The brains were separated into two hemispheres. Tissues from regions of the right hemisphere were collected for RT-qPCR analysis and storage at −80 °C. Fifteen different regions were dissected: the (1) pituitary gland, (2) the olfactory bulb, (3) substantia nigra, (4) medulla oblongata, (5) pons, (6) anterior part of the cerebellum, (7) motor cortex medial, (8) sensory cortex, (9) frontal basal cortex, (10) hippocampus, (11) caudate nucleus, (12) hypothalamus, (13) globus pallidus, (14) putamen, and (15) thalamus. In macaques, the pituitary gland weighs approximately 70 mg; for practical reasons, we preserved this tissue only for histological examination. For histological analysis, the left hemisphere, the pituitary gland, and one olfactory bulb were fixed in 10% neutral buffered formalin solution for 48 h and cryoprotected in 30% *w*/*v* sucrose in PBS. The cerebrum was dissected in 12 different coronal parts, cut at the anterior-posterior axis at +10, +8, +5, +1, −3, −6, −8, −11, −14, −18, and −22 mm from the bregma [21]; the cerebellum and pons were cut in four parts and embedded in paraffin before multiple consecutive 4 μm sections were prepared for immunohistochemical staining.

### 2.4. Viral RNA Detection in Brain Tissue

Brain tissue samples from the right hemisphere were homogenized [18]. After centrifuging, 100 μL supernatant was used for RNA isolation using a QIAamp Viral RNA Mini kit (Qiagen Benelux BV, Venlo, The Netherlands), following the manufacturer’s instructions. Viral RNA was reverse-transcribed to cDNA using a Transcriptor First Strand cDNA Synthesis kit (Roche Diagnostics BV, Almere, The Netherlands). Viral RNA was quantified by RT-qPCR specific for RdRp gene of SARS-CoV-2 [22]. Viral sub-genomic messenger RNA was detected, essentially, as described by Wölfel et al. [23]. For both assays, RNA standard curves were generated by in vitro transcription of the target regions. The assays had a limit of quantification of 20 RNA copies per reaction.

### 2.5. Immunohistochemistry

Deparaffinized tissue sections were stained as follows:

#### 2.5.1. CD3 and CD20

For antigen retrieval, slides were steamed for 1 h, in combination with IHC-Tek epitope retrieval solution (IHC world). Endogenous peroxidase activity was inhibited using PO blocking solution (S2023, DAKO) for 15 min. After washing, slides were incubated for 10 min in avidin (X0590, DAKO) and subsequently washed and incubated for 10 min with biotin (DAKO) to block endogenous biotin. Next, the slides were incubated for 20 min with PBS containing 0.1% BSA and 1% normal human serum and incubated overnight at 4 °C with the primary antibodies CD3 (polyclonal rabbit—anti-human CD3 IgG, cat. no. A045201-2, Agilent Technologies; 1:60 diluted) or CD20 (monoclonal mouse—anti-human CD20 IgG2a, clone L26, Agilent Technologies; 1:800 diluted). After washing, the slides were incubated with the secondary antibody (Rabbit-anti-mouse IgG biotinylated (E0354), Agilent Technologies; 1:200 diluted in PBS + 1% BSA + 1% NHS) and subsequently with Vectastain ABC peroxidase (ABC-PO, from Vector Laboratories; PK-4000; diluted 1:100 in PBS) for 30 min. After washing, 3,3′-diaminobenzidine (DAB) with 0.02% H_2_O_2_ was added for 20 min to visualize the antigen–antibody binding.

#### 2.5.2. Mamu-DR Staining

After epitope retrieval (as described for CD3 and CD20), the EnVision™ staining kit (G|2 double-stain system, rabbit/mouse, DAB+/permanent RED code K5361; Agilent technologies, Glostrup, Denmark) was used, in combination with Mamu-DR (monoclonal mouse–anti-human HLA-DR/DQ-IgG1, clone CR3/43, Agilent Technologies; 1:150 diluted).

#### 2.5.3. α-Synuclein

Epitope antigen retrieval was performed by 15 min incubation in 10% formic acid. After two washing steps, in PBS with 0.05% Tween, endogenous peroxidase and biotin activity were blocked, as described for CD3 and CD20. Non-specific antibody binding was prevented by 30 min incubation in PBS with 0.1% BSA, 1% NHS, and 0.02% Triton-X100. Next, the slides were incubated overnight at 4 °C with the primary antibody (clone 4D6, Biolegend) 1:200 diluted in 0.1% BSA in PBS. Secondary antibody and visualization were the same as used for CD3 and CD20.

#### 2.5.4. SARS-CoV-2

For viral antigen staining, a Roche Optiview DAB IHC kit, in combination with an anti-SARS-CoV-2-nucleoprotein (clone E16C; ThermoFisher), was used in a Ventana Benchmark Ultra immunostainer (Roche, Basel Switzerland), as previously described [7]. In brief, antigen retrieval took place with cell conditioning 1 (CC1, Ventana Medical Systems) (pH 8.5) for 24 min at 100 °C, 1/5.000 diluted. Thereafter, incubation took place with the primary antibody for 48 min at 36 °C, followed by standard Optiview detection/visualization with DAB and Copper.

After immunohistochemical staining, the sections were dehydrated and mounted with TissueTek^®^ coverslipping film (Sakura Finetek Europe B.V., The Netherlands). Hematoxylin-eosin (HE) staining was used for general morphology. Cells were counted in a blind manner. As a positive control, a lung section of a deceased COVID-patient was included (Figure S1).

## 3. Results and Discussion

We performed longitudinal ^18^F-FDG PET-CT analysis of the brains of the macaques that were featured in a SARS-CoV-2 infection study (Table 1) [18]. The uptake of the radioactively-labeled tracer ^18^F-FDG is widely used as a proxy for metabolic activity. ^18^F-FDG uptake is caused by natural processes that require glucose, even at rest, such as in the brain, myocardium, and intestine, as well as during oncogenesis and, importantly, inflammatory processes and infectious diseases [24]. This natural uptake causes an increased signal in the brain, which is further increased by using anesthetics during scanning. The latter may have an impact on glucose metabolism [25].

**Figure 1:**
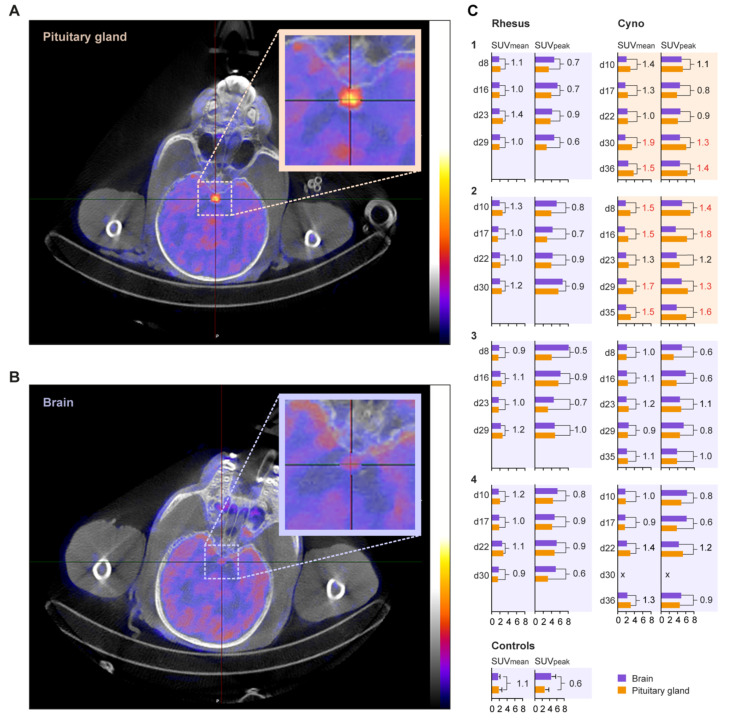
^18^F-FDG uptake in the pituitary gland after SARS-CoV-2 infection. Representative transversal slices of cynomolgus macaque C2, with increased ^18^F-FDG uptake in the pituitary gland, (**A**) and C3, with normal pituitary ^18^F-FDG uptake, (**B**) on day 8 are shown. The pituitary gland is indicated by the cross-hairs and boxed. Similar window-level settings are applied for both sections. (**C**) The standardized uptake values (SUV) of the brain and pituitary gland of each animal, per timepoint, are shown. On the *Y*-axis, the day post-infection of PET-CT and on the *X*-axis the SUV of the brain (purple bars) and pituitary gland (orange bars) are visualized for rhesus (left column) and cynomolgus (right column) macaques. For each animal, the SUV_mean_ and SUV_peak_ are shown. The numbers in the graph represent the ratio of the SUV for the pituitary gland value divided by the SUV of the brain. A deviation from normal is defined as SUV_mean_ ratio ≥ 1.5 and SUV_peak_ ratio ≥ 1. These ratios are indicated in red. Six non-infected animals were used as control for the definition of normal pituitary gland and brain SUVs. The animals boxed with an orange background showed increased uptake in the pituitary gland, while the animals boxed with a purple background do not show this increase.

Despite the background activity, increased tracer uptake was observed in the pituitary gland of cynomolgus macaques C1 and C2 (Figure 1A), but not in the other animals (Figure 1B). Under normal physiological conditions, the metabolic activity of the pituitary gland is comparable to the surrounding tissue (Figure 1B) [26,27]. Since the quantitative values of ^18^F-FDG uptake can be affected by multiple biological and physical factors [28], the normal pituitary gland–brain ratio of the standardized uptake values (SUVs) was determined in six non-infected control macaques (Figure 1). In most SARS-CoV-2-infected macaques the SUV_mean_ and SUV_peak_ were not significantly elevated, compared to the uninfected control macaques. In macaque C1, however, the pituitary gland–brain ratio was elevated on 30- and 36-days post-infection, with SUV_mean_ ratios of 1.9 and 1.5 and SUV_peak_ ratios of 1.3 and 1.4, respectively. This was also observed in macaque C2 on days 8, 16, 29, and 35 pi, with SUV_mean_ ratios between 1.5–1.7 and SUV_peak_ ratios between 1.3–1.8 (Figure 1C).

**Figure 2:**
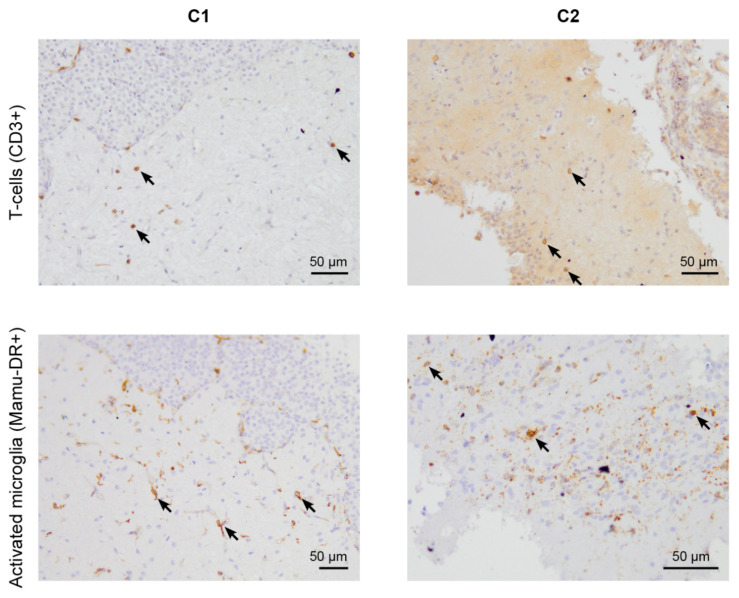
Immunohistochemistry for T-cells and activated microglia (some positive cells are indicated with arrows) of SARS-CoV-2-infected brain sections of cynomolgus macaques C1 and C2. The presence of T-cells (CD3+) (top level) and activated microglia cells (Mamu-DR+) (bottom level) in the pituitary gland of monkeys C1 (**left**) and C2 (**right**), observed five weeks after infection.

To investigate whether the increased accumulation of tracer coincided with either the presence of SARS-CoV-2 or neuroinflammation, we performed immunohistochemistry in the pituitary gland of the animals. Analysis showed an infiltration of T-cells and activated microglia cells in the pituitary gland of animals C1 and C2 (Table 2, Figure 2). These results hint towards immune activation. However, no infiltrated B-cells or viral antigens were detected in the pituitary gland. The finding of immune activation, in the absence of a viral antigen, is explainable. The animals were sacrificed on day 36 and 35 pi, respectively. The virus could have been initially present in the brain at an earlier time point but was cleared in a later instance. Moreover, these data are in line with several reports describing immune activation in the brains of human patients who succumbed to COVID-19, without detecting viral RNA or antigen [7,8].

The increased uptake of ^18^F-FDG in the pituitary gland may less likely be a direct effect of infected tissue, but more likely due to changes on the hypothalamic-pituitary-adrenal axis, as an increased uptake is, in all cases, the result of an abnormality in humans [29,30]. The impact of a SARS-CoV-2 infection on the hypothalamic-pituitary-adrenal axis has been investigated in humans, and differences in cortisol levels between infected and healthy people were found. This suggests that both hypercortisolism, similar to what we found in the macaques, and hypocortisolism can be associated with a SARS-CoV-2 infection. However, until now, the effects of an up- or downregulation on disease severity is not proven [31,32].

Table 2 outlines the presence of (1) T-cells (CD3+ cells) in the brain tissue (intraparenchymal),around blood vessels (perivascular), in group formation (nodules), or in the meninges; (2) activated microglia (Mamu-DR+ cells) in different parts of the brain, the morphology as a measure for the severity of activation (ramified or amoeboid), in group formation (nodules), or in the meninges; (3) α-synuclein/Lewy bodies (α-synuclein + cells with inclusions) in the ventral midbrain region, next to the caudate nucleus. White squares represent 0 positive cells, light grey: 1–5 positive cells (light), dark grey: 5–10 positive cells (moderate), black > 10 positive cells (moderate to severe), per slice, by a 200× magnification.

**Figure 3:**
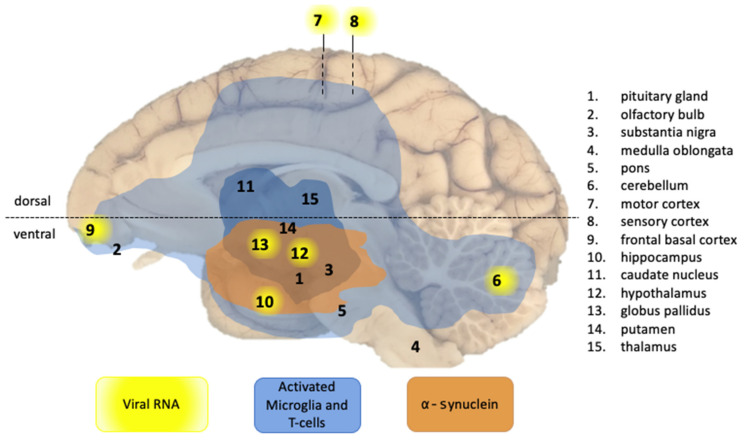
Overview of CNS effects of SARS-CoV-2 infection detected in a macaque brain in this study. The presence of viral RNA was investigated in multiple regions of the brain, as indicated by the numbers. Viral RNA-positive regions in cynomolgus macaque C3 are indicated by a yellow background. Brain areas with infiltrated T-cells (CD3+) and activated microglia (Mamu-DR+) are shown in light blue (low expression) and dark blue (moderate expression). Brain areas with Lewy bodies (α-synuclein+) are depicted in orange. Blue and orange areas comprise of the combined observations from all animals. The horizontal dotted line indicates the border between the dorsal and ventral parts of the brain.

Next, we systematically examined the brains of all SARS-CoV-2-infected animals in this study for the presence of viral genetic material in 15 different brain regions (Figure 3), as well as signs of virus-induced neuroinflammation. Of the eight macaques studied, viral RNA was only detected in the brain of cynomolgus macaque C3. More precisely, the cerebellum (1.48 × 10^5^ genome equivalents (GE)/gram), medial motor cortex (2.09 × 10^5^ GE/gram), sensory cortex (2.07 × 10^5^ GE/gram), and frontal basal cortex (8.29 × 10^4^ GE/gram), as well as hippocampus (1.24 × 10^5^ GE/gram), hypothalamus (1.05 × 10^6^ GE/gram), and globus pallidus (5.45 × 10^4^ GE/gram) tested positive in the RT-qPCR. No viral RNA was detected in the samples collected from the olfactory bulb, substantia nigra, medulla oblongata, pons, caudate nucleus, and putamen. Notably, a small cerebral infarction was also observed in the U-fibers of the midbrain in animal C3 (Table 2). We found no evidence for active virus replication in the brains of any of the SARS-CoV-2-infected macaques at the time point of euthanasia, as the subgenomic messenger-RNA analysis was negative. In line with this, SARS-CoV-2 antigen was not detected by immunohistochemistry in the postmortem samples.

**Figure 4:**
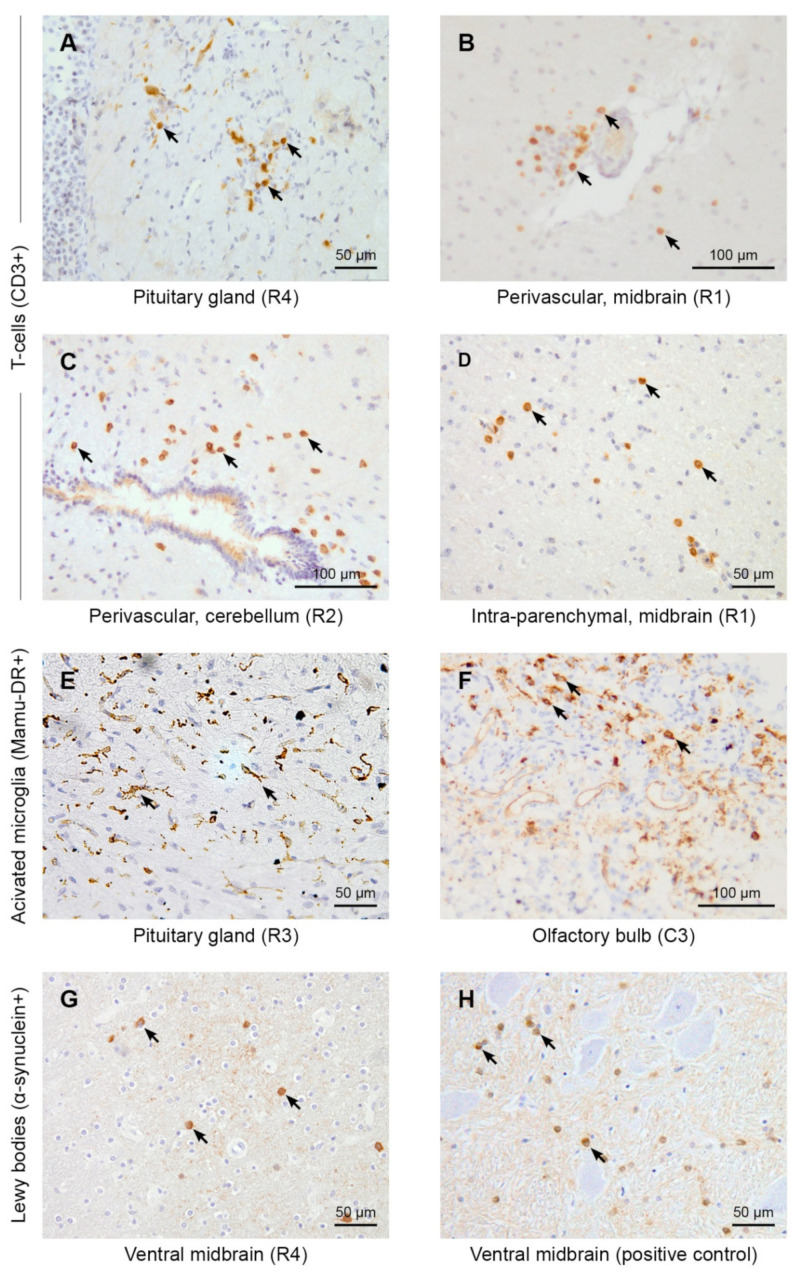
SARS-CoV-2 causes brain inflammation and Lewy body formation in brains of macaques. The immunohistochemistry of macaque brain tissues (20×). Arrows indicate the presence of some clear positive cells for the immunohistochemical staining. First and second row: CD3+ T-cells. In infected animals, T-cells were found in the pituitary gland (**A**), perivascular (**B**,**C**), and in the brain parenchyma (**D**). Third row: Mamu-DR+-activated microglia cells. Activated microglia cells are shown in the pituitary gland of R3 (**E**). Amoeboid microglia cells are shown in the olfactory bulb of C3 (**F**). Bottom panel: α-Synuclein positive staining was found in the ventral midbrain in all SARS-CoV-2-infected rhesus macaques. α-Synuclein accumulations were found in the ventral midbrain region, next to the caudate nucleus (**G**). A positive control of the α-synuclein staining of a brain slice from a 22-year-old cynomolgus monkey, showing signs of parkinsonism from the brain bank of the BPRC, is shown (**H**).

Nevertheless, a neuroinflammatory response to a SARS-CoV-2 infection was observed in all infected animals, both rhesus and cynomolgus macaques, which was absent in the control animals (Table 2, Figure 4).

As observed in cynomolgus macaques C1 and C2, T-cell infiltration in the pituitary gland was documented in most animals (Figure 4A), except for rhesus macaque R1. However, R1 did show perivascular T-cells throughout the brain (Figure 4B), which was also seen in most other infected animals (Figure 4C). CD3 staining manifested infiltrated T-cells in the brain parenchyma in the majority of the animals (Figure 4D), but rarely showed T-cells aggregated in small nodules. Moreover, meningeal T-cells were observed in only two rhesus macaques (R2 and R3).

Activated microglia, made visible by Mamu-DR staining, were present in all infected animals in various brain regions, including the pituitary gland (Figure 4E) and olfactory bulb (Figure 4F). When analyzing the morphology of these Mamu-DR+ microglia, most displayed a ramified morphology (Figure 4E); however, in the olfactory bulb of animal C3, an amoeboid morphology was also observed (Figure 4F). Nodules of microglia cells were rarely seen, but meningeal expression of Mamu-DR was present in all infected animals. Additionally, no activation of astrocytes was observed by GFAP staining in the brain of all monkeys (Figure S2). Additionally, no general abnormalities were found with the HE-stain in the brain of virus-exposed monkeys, including the absence of ischemic/necrotic lesions or areas with loss of myeline.

None of the control rhesus macaques displayed obvious signs of activation of microglia or T-cells. The control cynomolgus macaques showed only a minimal presence of T-cells (pituitary gland of C5) and few activated microglia cells (pituitary gland of C6).

Several case studies reported parkinsonism two to five weeks after SARS-CoV-2 infection [3,4,5,6]. The risk of neuropathology, induced by SARS-CoV-2, may be a more general phenomenon linked to coronaviruses, as such the complications that have been described after the outbreaks of closely related SARS and MERS coronaviruses [33,34]. Moreover, it has been hypothesized that certain viruses can induce neurological manifestations, including parkinsonism [35]. The formation of Lewy bodies, a hallmark of parkinsonism, represents intracellular accumulations of α-synuclein and is often found around the substantia nigra, a dopaminergic nucleus in the midbrain region. For that reason, we screened the ventral midbrain regions of all animals for α-synuclein. Intracellular α-synuclein accumulations were detected in Lewy body-like structures, but no Lewy neurites, in all infected rhesus macaques and one cynomolgus macaque, C4 (Table 2, Figure 3 and Figure 4G,H). The α-synuclein accumulations were absent in the brains of all four control animals. For animal C4, we cannot exclude an age-related factor to parkinsonism, as this animal was older (16 years) than the other animals (5–7 years). Staining for amyloid plaques (Campbell-Switzer and amyloid-beta_39–43_ immunohistochemistry) [36] revealed no amyloid accumulation in the ventral midbrain sections of any of the animals, indicating that the α-synuclein accumulations were not caused by general protein aggregation processes.

The data from this animal study are indicative for a SARS-CoV-2-induced activated immune landscape in the brain occurring in the post-acute phase of infection, 2–5 weeks post exposure. So far, brain inflammation has only been observed in patients deceased after severe COVID-19 [7,8]. Limited data are available of brains from people who suffered from mild-to-moderate COVID-19. Functional magnetic resonance imaging (fMRI) of brains from these people suggested longitudinal effects in different brain areas [37]. In addition, a recent report on divergent immune responses between CSF and peripheral blood in hospitalized patients, with neurological complications, implies SARS-CoV-2 can also affect the CNS during non-lethal infection [38].

Here, we describe the detection of intracellular α-synuclein aggregates in the brains of SARS-CoV-2-infected macaques and, moreover, provide evidence for a broad spectrum of inflammatory processes in the brain. The animals showed no overt clinical signs, yet medical imaging techniques showed mild-to-moderate pulmonary disease [18]. Detection of viral RNA in the brain of one animal demonstrates the virus’ neuroinvasive capability. This matches a study describing SARS-CoV-2 neuroinvasion in mouse brains and human brain organoids [38]. More importantly, our data are in line with the finding by Yang et al., who described the presence of infiltrating T-cells in the brain parenchyma, as well as the activation of microglia cells in patients, after lethal COVID-19 [7,8].

There is a growing concern about the long-term consequences of asymptomatic, mild, or moderate SARS-CoV-2 infection in humans. Our data highlight the potential of the virus to cause pathology in the brain of macaques. In all eight infected animals, we observed a range of neurological abnormalities, such as a hypermetabolic pituitary gland, α-synuclein inclusions, activated microglia in the brain parenchyma, and infiltrating T-cells (Figure 4).

To what extent our observations in the translational macaque model for COVID-19 are predictive for SARS-CoV-2-induced neuropathology in humans is currently unclear. However, the findings can be regarded as a warning for the risk of developing long-term neurological complications, even after an asymptomatic infection or mild disease process.

## Figures and Tables

**Table 1:** Animals used in the study.

	Code	Age (years)	Weight (kg)	Cum VL ^1^	Cum CT ^2^	Shedding (dpi) ^3^	Seroconversion (dpi) ^4^	Euthanasia (dpi)
**Rhesus**								
R14002	R1	6	8.2	2.10 × 10^7^	12	1–10	16	36
R15080	R2	5	7.9	2.69 × 10^6^	8	1–4	16	35
R15090	R3	5	7.8	4.64 × 10^5^	4	1–5	16	36
R15096	R4	5	8.7	1.70 × 10^4^	22	1	16	35
**Cynomolgus**								
J16004	C1	4	5.7	1.28 × 10^5^	14	1–4	12	42
J16012	C2	4	3.3	3.00 × 10^5^	30	1–2	12	38
J16017	C3	4	4.9	1.92 × 10^8^	9	1–6	12	38
Ji04080	C4	16	9.7	1.19 × 10^5^	18	1–3	17	42

^1^ Cumulative virus load. Sum of virus loads measured at days 0, 2, 4, 6, 8, 10, 12, 14, 16, and 22 pi. ^2^ Cumulative CT scores; sum of the CT scores of the lungs of each timepoint were measured at days 0, 2, 4, 6, 10, 12, 14, 16, 22, and 30 pi, as well as the day of euthanasia. A maximum of 30 could be scored at each timepoint. ^3^ Period of virus shedding from throat indicated are the first to last day of detection. ^4^ Day after infection with first observation of virus-specific IgG antibodies [18].

**Table 2:** Histological findings.

Marker		Brain Area	Rhesus				Cynomolgus				Controls							
			R1	R2	R3	R4	C1	C2	C3	C4	R5	R6	C5	C6				
**T-cells (CD3+)**	intra-parenchymal	pituitary gland																
		olfactory bulb																
		front brain																
		dorsal																
		ventral							⊙									
		cerebellum																
	perivasculair	pituitary gland																
		olfactory bulb																
		front brain																
		dorsal																
		ventral																
		cerebellum																
	nodules	pituitary gland																
		olfactory bulb																
		front brain																
		dorsal																
		ventral																
		cerebellum																
	meninges	pituitary gland																
		olfactory bulb																
		front brain																
		dorsal																
		ventral																
		cerebellum																
**Activated microglia (Mamu-DR+)**	presence	pituitary gland																
		olfactory bulb																
		front brain																
		dorsal																
		ventral																
		cerebellum																
	morphology: ramified/ amoeboid	pituitary gland																
		olfactory bulb																
		front brain																
		dorsal																
		ventral																
		cerebellum																
	nodules	pituitary gland																
		olfactory bulb																
		front brain																
		dorsal																
		ventral														**Observation of indicated marker:**		
		cerebellum																
	meninges	pituitary gland																light (inlc. ramified microglia)
		olfactory bulb																
		front brain																moderate (incl. amoeboid microglia)
		dorsal																
		ventral																moderate to severe
		cerebellum																
**α-synuclein+**	inclusions	ventral midbrain														⊙		infarction

## Data Availability

All data are available in the main text or Supplementary Materials. Correspondence and request for materials should be addressed to E.J.V. (verschoor@bprc.nl).

## Supplementary Materials

The following supporting information can be downloaded at https://www.mdpi.com/article/10.3390/v14040776/s1. Table S1: Control animals used in the study. Figure S1: Lung section of a deceased COVID-patient, who was used as positive control for viral antigen staining. Figure S2: Examples of a GFAP staining for astrocytes from the dorsal midbrain area of an (A) infected rhesus macaque and (B) uninfected control monkey. Activation of astrocytes was observed neither in the SARS-CoV-2-infected monkeys nor the controls.
