# Supplementary material for: Brain Inflammation and Intracellular α-Synuclein Aggregates in Macaques after SARS-CoV-2 Infection
Source: Viruses. 2022 Apr 8;14(4):776. doi: 10.3390/v14040776 (PMC9025893; doi:10.3390/v14040776)
Supplement: Supplementary file 1 [file viruses-14-00776-s001.zip › Table S1.pdf]

Histology control animals

animal	code	age (yrs)	weight [kg]
R11084	R5	5	8.5
R12017	R6	6	5.1
J15018	C5	5	5.3
J13004	C6	7	5.1

PET-CT control animals

animal	code	age (yrs)	weight [kg]
R11074	R7	9	8.3
R14129	R8	6	5.9
R12058	R9	8	8.3
R13091	R10	7	8.1
R14004	R11	6	6.8
R14006	R12	6	6.7
