# Supplementary figures and images for: Brain Inflammation and Intracellular α-Synuclein Aggregates in Macaques after SARS-CoV-2 Infection
Source: Viruses. 2022 Apr 8;14(4):776. doi: 10.3390/v14040776 (PMC9025893; doi:10.3390/v14040776)

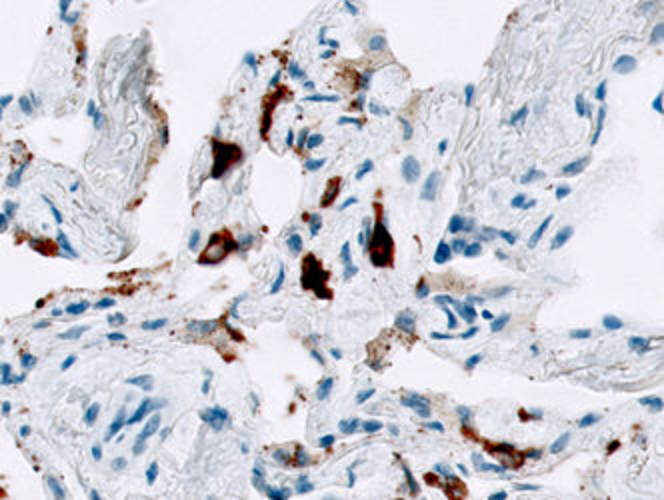

Supplement: Supplementary file 1 [file viruses-14-00776-s001.zip › fig S1.pdf]

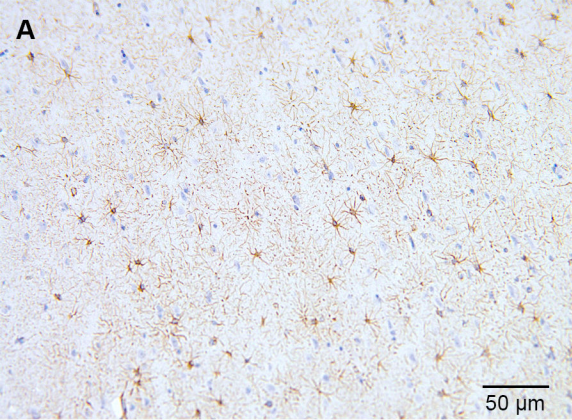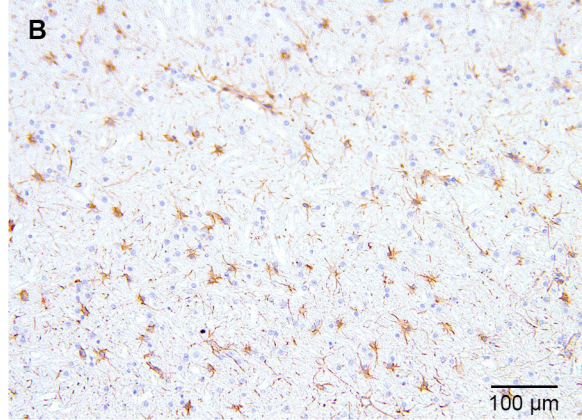

Supplement: Supplementary file 1 [file viruses-14-00776-s001.zip › fig S2.pdf]
